# Investigation of Maximum Monosyllabic Word Recognition as a Predictor of Speech Understanding with Cochlear Implant

**DOI:** 10.3390/jcm13030646

**Published:** 2024-01-23

**Authors:** Ronja Czurda, Thomas Wesarg, Antje Aschendorff, Rainer Linus Beck, Thomas Hocke, Manuel Christoph Ketterer, Susan Arndt

**Affiliations:** 1 Department of Otorhinolaryngology, Medical Center—University of Freiburg, Faculty of Medicine, University of Freiburg, Killianstr. 5, 79106 Freiburg, Germany; thomas.wesarg@uniklinik-freiburg.de (T.W.); antje.aschendorff@uniklinik-freiburg.de (A.A.); rainer.beck@uniklinik-freiburg.de (R.L.B.); manuel.christoph.ketterer@uniklinik-freiburg.de (M.C.K.); susan.arndt@uniklinik-freiburg.de (S.A.); 2 Cochlear Deutschland GmbH & Co., KG, Mailänder Straße 4 a, 30539 Hannover, Germany; thocke@cochlear.com

**Keywords:** cochlear implant, speech audiometry, word recognition, hearing loss, hearing aid, maximum word recognition

## Abstract

**Background:** The cochlear implant (CI) is an established treatment option for patients with inadequate speech understanding and insufficient aided scores. Nevertheless, reliable predictive models and specific therapy goals regarding achievable speech understanding are still lacking. **Method:** In this retrospective study, 601 cases of CI fittings between 2005 and 2021 at the University Medical Center Freiburg were analyzed. We investigated the preoperative unaided maximum word recognition score (mWRS) as a minimum predictor for post-interventional scores at 65 dB SPL, WRS_65_(CI). The WRS_65_(CI) was compared with the preoperative-aided WRS, and a previously published prediction model for the WRS_65_(CI) was reviewed. Furthermore, the effect of duration of hearing loss, duration of HA fitting, and etiology on WRS_65_(CI) were investigated. **Results:** In 95.5% of the cases, a significant improvement in word recognition was observed after CI. WRS_65_(CI) achieved or exceeded mWRS in 97% of cases. Etiology had a significant impact on WRS_65_(CI). The predicted score was missed by more than 20 percentage points in 12.8% of cases. **Discussion:** Our results confirmed the minimum prediction via mWRS. A more precise prediction of the expected WRS_65_(CI) is possible. The etiology of hearing loss should be considered in the indication and postoperative care to achieve optimal results.

## 1. Introduction

In recent decades, the cochlear implant (CI) has become an established treatment option for patients with severe to profound hearing loss or impairment, for whom the fitting of a hearing aid (HA) or other hearing amplification measures no longer ensure adequate speech understanding [1,2,3,4,5]. Progressive improvements in surgery, technology, and rehabilitation measures have led to a constant expansion of the indication criteria [6,7,8,9,10,11], so that, since 2020, the S2k guideline in Germany recommends cochlear implantation of a patient from the point of monosyllabic word recognition of ≤60% at a sound level of 65 dB SPL after optimized HA fitting [2]. This specific value for the audiological indication has not yet been matched by a value of speech understanding that should be achieved postoperatively in German-speaking countries. According to the guidelines for cochlear implant treatment of the German Society of Oto-Rhino-Laryngology, an improvement in speech understanding of at least 20 percentage points with cochlear implantation can be expected [5]. In addition, initial studies highlight preoperative maximum word recognition (mWRS) as a suitable individual predictor for postoperative word recognition with CI at 65 dB SPL [12,13,14], referred to as WRS_65_(CI) in the following. In the typical German clinic population of the last decade, approximately 96% of patients can be assumed to have a WRS_65_(CI) that achieved or exceeded the preoperative mWRS [12].

Model approaches are available for the expected outcomes of care, which appear suitable for determining an expectation corridor for a patient population [15,16], which aims to provide individual predictions of expected postoperative WRS [17,18]. Hoppe et al. [17] have so far achieved a prediction error in the order of 11–14 percentage points (pp) with their model for sample sizes of about 100 patients with preoperatively measurable mWRS [19]. In this model, mWRS, monosyllabic word recognition with a HA at 65 dB SPL, WRS_65_(HA), and the patient’s age at the time of CI surgery are taken into account according to Equation (1):

(1) $${\mathrm{WRS}}_{65}(\mathrm{CI})\;[\%]=\frac{100}{1+e^{-({ß}_{0}+{ß}_{1}\cdot mWRS+{ß}_{2}\cdot Age+{ß}_{3}\cdot {WRS}_{65}(HA))}}$$

where ß_0_ = 0.84 ± 0.18, ß_1_ = (0.012 ± 0.0015)/%, ß_2_ = (−0.0094 ± 0.0025)/years, and ß_3_ = (0.0059 ± 0.0026)/%.

However, this prediction is limited to patients with a preoperative mWRS greater than zero [19]. Until now, factors such as etiology, duration of hearing loss, duration of HA use, and duration of untreated hearing loss have not been taken into account in relation to the equation, and insufficient case numbers have also been problematic [17,19].

The aim of this study was to evaluate both approaches: the minimum prediction of the WRS_65_(CI) based on the mWRS [12] and the model according to Equation (1). Both result “in a corridor within which the postoperative word recognition score with CI should be” [19], in the largest group of patients (to our knowledge) with a mWRS greater than 0%. The influence of etiology, duration of hearing loss, and duration of HA fitting on WRS_65_(CI) and the deviation from the prognosis according to Equation (1) was additionally investigated.

## 2. Materials and Methods

The present retrospective study was performed with the approval of the Ethics Committee Freiburg (EK-Freiburg: 23-1029-S1-retro) (DRKS00029966) and in compliance with national law and the Declaration of Helsinki of 2013 (in the current revised edition).

### 2.1. Patients

The present data were collected between January 2005 and December 2021 within the framework of CI pre-evaluation and during basic and follow-up therapies of CI care in the Department of Otolaryngology at the University Medical Center Freiburg. The inclusion criteria were defined as uni- or bilateral implantation, age over 18 years at the time of implantation, measurable preoperative unaided monosyllabic word recognition in the implanted ear greater than 0%, together with available data on preoperative speech understanding with HA, CI experience of at least 6 months, and completed CI rehabilitation in Freiburg. Data from patients with neurological or psychiatric concomitant diseases relevant to speech understanding were excluded. Medical history data included age, gender, duration of subjective hearing loss, and etiology.

**Figure 1:**
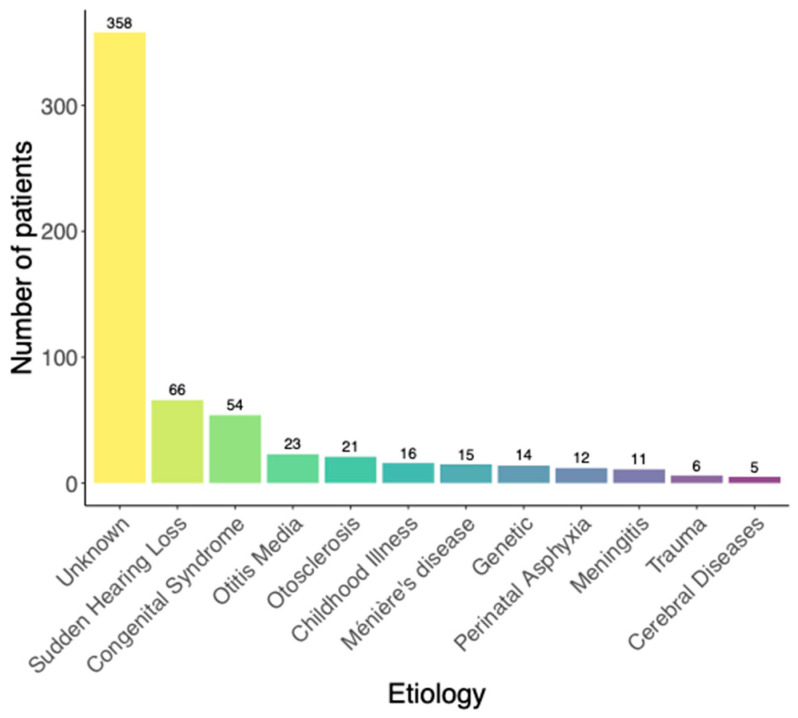
Distribution of etiologies of hearing loss.

A total of 601 ears (cases) from 531 patients, 70 of whom were fitted bilaterally with CI, were identified to meet the inclusion criteria and were included. The demographic distribution of this study population is summarized in Table 1. The information on the duration of hearing loss and HA usage was collected through a questionnaire and is based on the subjective assessment of the patients. Figure 1 presents the distribution of etiologies of hearing loss. The “childhood disease” category includes mumps, measles, and rubella. Causes such as acoustic neuroma, medulloblastoma, and superficial siderosis have been grouped into cerebral diseases.

### 2.2. Audiometry

Hearing loss in air conduction was averaged over the four octave frequencies (500, 1000, 2000, and 4000 Hz) and is reported here as a four-frequency-pure tone average (4PTA). The hearing thresholds were measured with headphones in a soundproof room for each ear separately. The opposite ear was masked, if necessary. For hearing thresholds exceeding the performance limit of the audiometers, a value of 120 dB HL was used in the analysis.

Speech understanding was assessed as word recognition in silence using the Freiburg monosyllabic test. Preoperatively, the mWRS and the WRS_65_(HA) were measured. Postoperatively, the WRS_65_(CI) was assessed after a period of at least six months after initial fitting.

### 2.3. Data Analysis

The statistical analysis of the data and the creation of the figures were carried out with R (R version 4.2.1; R Core Team (2023). R: A language and environment for statistical computing. R Foundation for Statistical Computing, Vienna, URL. https://www.R-project.org/ accessed on 15 February 2023).

To check for a normal distribution, the data sets were assessed using a Q-Q plot.

We performed statistical analysis to investigate the impact of (1) duration of hearing loss and (2) etiology.

(1) To investigate the impact of duration on hearing loss, we applied unpaired *t*-tests. Using the unpaired *t*-test, we compare the mean values of WRS_65_(CI) between the group with a hearing loss > 20 years and the group with a hearing loss ≤ 20 years.
(2) The effect of etiology on WRS_65_(CI) was analysed using a Kruskal–Wallis test. To further investigate the impact of etiology on postoperative outcome, post hoc comparisons were made between the various causes of severe to profound hearing loss using Dunn’s test. To correct for multiple testing, a Holm adjustment was applied.

Missing data were not imputed. Cases with missing preoperative aided scores were excluded from model calculations according to Equation (1) but used for the evaluation of the minimum prediction via mWRS.

## 3. Results

### 3.1. Preoperative Results

**Figure 2:**
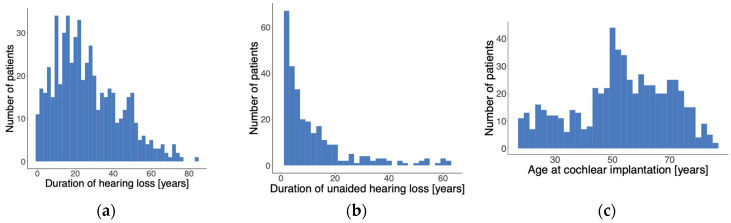
Patient characteristics. (**a**) Distribution of duration of hearing loss. (**b**) Distribution of duration of unaided hearing loss. (**c**) Distribution of age at cochlear implantation.

Figure 2 illustrates the distribution of the duration of hearing loss and age at cochlear implantation and the duration of unaided hearing loss [duration of hearing loss − duration of hearing aid fitting]. The duration of hearing loss was defined retrospectively as the duration between the anamnestic onset and the time of the preoperative assessment of this loss.

On average, patients reported a duration of hearing loss of 26.3 ± 17.0 years and a duration of HA fitting of 20.4 ± 14 years. Data were missing in 62 cases.

Based on the calculation of the duration of unaided hearing loss, an unaided period of >10 years was determined for 111 ears. Of these, 46 cases had unaided hearing loss for more than 20 years.

On average, the cases with unaided hearing loss for >20 years (*n* = 46) achieved a mean WRS_65_(CI) of 68.6% ± 25.0%, whereas the cohort with untreated hearing loss for ≤20 years (*n* = 493 cases) achieved 74.2% ± 20.4%. The WRS_65_(CI) was 74.0% ± 21.0% for the entire study population (*n* = 601).

(1) Applying an unpaired *t*-test between subjects with a duration of hearing loss >20 years and those with ≤20 years, we found no significant difference in the WRS_65_(CI) (*p* > 0.05).

**Figure 3:**
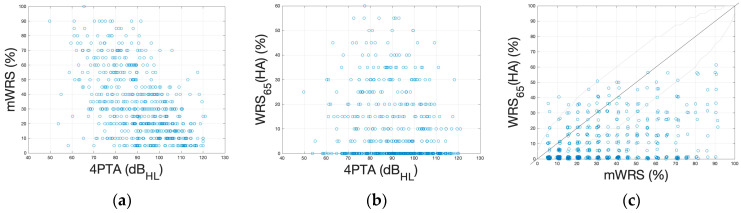
Scatterplots of pre- and postoperative word recognition in relation to different preoperative measurements. (**a**) Four-frequency pure-tone average, 4PTA, versus mWRS; (**b**) 4PTA versus WRS_65_(HA). (**c**) Preoperative mWRS versus WRS_65_(HA). The boundaries around the bisectors represent the critical differences, according to Winkler and Holube [20]. Points outside these limits can be interpreted as significant differences in the respective values.

Figure 3 illustrates the relationships between different preoperative measurements. Figure 3a shows the relationship between the 4PTA and the mWRS. Overall, 43 ears (7.2%) had a 4PTA of <70 dB HL. Among these, 20 ears (3.3%) only achieved an mWRS of ≤50%, despite showing a low 4PTA. One hundred ears showed a 4PTA between 70 and 80 dB HL, 144 between 80 and 90 dB HL, and 308 greater than 90 dB. In five cases, these measurements are missing. Figure 3b shows the WRS_65_(HA) versus the 4PTA, whereas Figure 3c plots the WRS_65_(HA) versus the mWRS.

The group results of the preoperative measurements are illustrated in Table 2.

### 3.2. Postoperative Results

**Figure 4:**
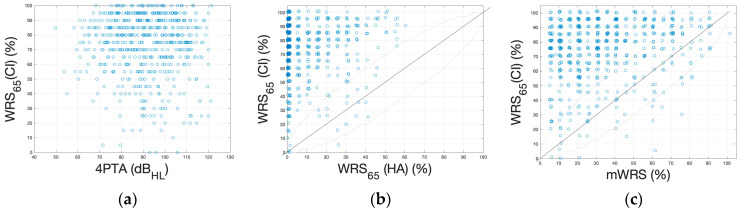
Scatterplots of postoperative word recognition in relation to preoperative measurements. (**a**) Postoperative WRS_65_(CI) versus preoperative four-frequency pure-tone average, 4PTA. (**b**) WRS_65_(CI) versus preoperative WRS_65_(HA). (**c**) WRS_65_(CI) versus preoperative mWRS. The boundaries around the bisectors represent the critical differences, according to Winkler and Holube [20]. Points outside these limits can be interpreted as significant differences in the respective values.

Figure 4a shows the relationship between the WRS_65_(CI) and the preoperative 4PTA, whereas Figure 4b relates the WRS_65_(CI) to the WRS_65_(HA). Figure 4c presents the relationship between the postoperative WRS_65_(CI) and the preoperative mWRS.

Until 2012, it was standard practice in our clinics, as in many other clinics in Germany, to measure monosyllabic word recognition with a HA at 70 dB SPL because of the otherwise often lack of speech recognition at lower sound pressure levels. Therefore, data concerning monosyllabic word recognition at 65 dB SPL with a HA were only available for 494 ears. Of these, 95.5% (*n* = 472) ears showed significantly improved word recognition with CI compared to word recognition with HA, both at 65 dB SPL. A significant deterioration [20] was only observed in one case (Figure 4b). The average speech understanding increases overall from an WRS_65_(HA) of 10% to an WRS_65_(CI) of 74%/65 dB. This corresponds to an improvement of 74%.

The scatterplot of the mWRS and the WRS_65_(CI) in Figure 4c shows that the mWRS was achieved or exceeded by the WRS_65_(CI) in 97% (*n* = 582) of cases. Thus, only 3% (*n* = 19) of cases yielded a WRS_65_(CI) below the minimum predictor for the outcome with CI.

### 3.3. Effect of Etiology on Postoperative Speech Understanding

**Figure 5:**
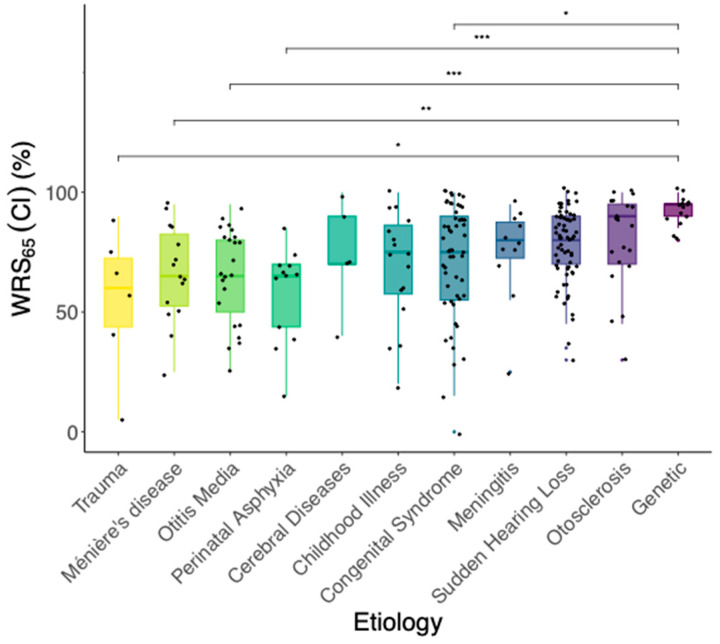
Box-whisker plots of WRS_65_(CI) for different etiologies of hearing loss. The order of the plots is based on the ascending median values from left to right. The density and dispersion of the data points demonstrate the frequency of each etiology and the distribution of postoperative outcomes. Data points represent individual ears. * represents *p* < 0.05, ** represents *p* < 0.01 and *** represents *p* < 0.001.

Figure 5 shows box-whisker plots of word recognition with CI for the different etiologies of hearing loss. In the comparisons of WRS_65_(CI) between the different etiologies, the group with an unknown cause of deafness was excluded from the analysis in order to identify specific and clinically relevant differences between the known etiologies.

(2) In the Kruskal–Wallis-Test, a statistically significant effect of the etiology of hearing loss on WRS_65_(CI) (χ^2^ = 36.75, *p* < 0.05) was found. Five out of the 55 pairwise comparisons of the etiologies (corrected with Holm) showed a significant difference in WRS_65_(CI). On the group median, cases with the etiology “Congenital”, “Trauma”, “Meniere’s disease”, “Otitis media” or “Perinatal asphyxia” revealed worse postoperative speech understanding compared to genetic hearing loss.

### 3.4. Validation of the Prediction Model

**Figure 6:**
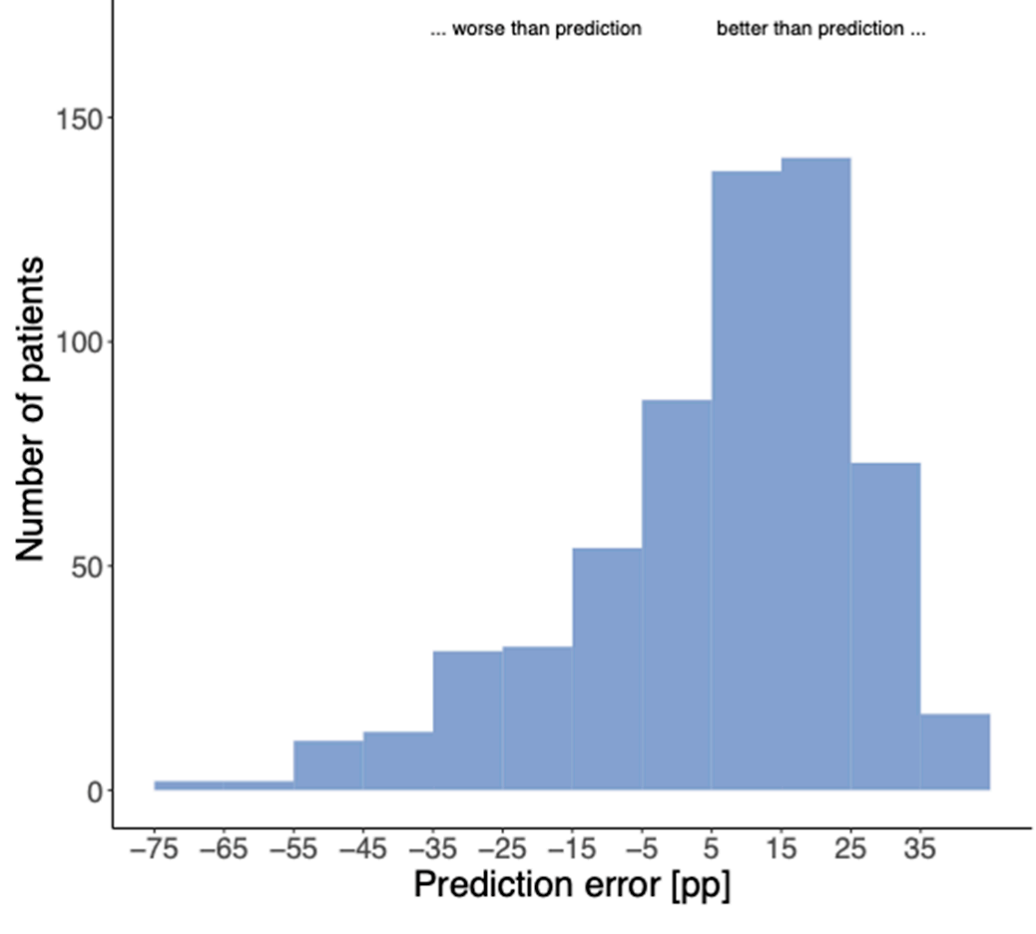
Frequency distribution of the differences between measured and predicted WRS_65_(CI) based on Equation (1). In all cases with negative values, the prediction was not achieved.

Figure 6 shows the frequency distribution of the prediction error calculated as the difference between the actual word recognition with CI after at least 6 months and the predicted word recognition according to Equation (1). In all cases with a positive difference, the predicted word recognition was exceeded, whereas all cases with a negative difference did not achieve the prediction. In the present population of 601 cases with a mWRS > 0%, the prediction was missed by more than 20 percentage points (pp) downward in 77 cases (12.8%).

The median absolute error of the prediction according to Equation (1) is 16.1 pp. Table 3 summarizes the effect of the etiology on selected location parameters of the distribution with respect to the results of the total population (left columns “Absolute” and ”Relative to Model”; the latter have been corrected by the above-mentioned 9.9 pp to improve the visualization of the lower dispersion attributable to etiologies). Cases with genetic hearing loss exhibited significantly better WRS_65_(CI) than the whole population. In contrast, cases with perinatal asphyxia showed significantly below-average WRS_65_(CI). Regarding model error, only cases with perinatal asphyxia were found to have significantly worse than predicted WRS_65_(CI). Overall, consideration of etiology with respect to the model leads to a significantly lower deviation from the prediction for the total population (sign test: *p* = 5 × 10^−4^, expressed by the model error). From the difference in *p*-values for WRS_65_(CI) and model error, it follows that preoperative mWRS and WRS_65_(HA) partly include the effect of etiology on WRS_65_(CI).

For some etiologies (perinatal asphyxia, Menière’s disease, genetic hearing loss, otitis media, trauma, and cerebral disease), interquartile ranges of model error greater than 30 pp can be identified. These are not equivalent to a worse prediction on average but indicate much greater variability that cannot be explained by the model within the corresponding patient groups.

## 4. Discussion

Of the 494 cases with available data on word recognition with HA at 65 dB SPL, 472 (95.5%) had significantly better speech understanding with CI at at least six months compared with preoperative HA, and one case showed a significantly poorer outcome. Overall word recognition improved by 64 pp to 74%.

The clinical relevance of maximum monosyllabic word recognition as a minimum outcome predictor was confirmed within this retrospective study in the largest patient population to date using the inclusion criterion of a preoperative mWRS greater than zero percent. In only 3% of the cases, the mWRS could not be achieved with CI.

The model for estimating the postoperative WRS_65_(CI) according to Equation (1) were confirmed by the data of our patients. The median absolute error of the prediction according to Equation (1) is 16.1 pp. This is a higher deviation than the 11 or 14 pp reported by Hoppe et al. [17,19]. However, the median error of 9.9 pp found in our study reveals that this higher absolute error can be justified by the overall result above prediction and, thus, by an even better result than predicted. The model according to Equation (1) refers to six-monthly values for the WRS_65_(CI), whereas six-month and later time points were analyzed in the present retrospective study.

As previously described in a very large patient collective with 2251 patients [15], etiology had a significant effect on postoperative speech understanding in the present study. For the subpopulation with a genetic cause of hearing loss, both studies found a relatively small but significant positive effect on WRS_65_(CI). In contrast, Blamey et al. [15] determined above-average results for Menière’s disease, whereas we report a median WRS_65_(CI) of 15 pp below the value for the total population for the included 15 cases. This might be attributable to one inclusion criterion. Whereas Blamey et al. probably included mainly cases without preoperative speech understanding, i.e., presumably with inactive Menière’s disease, we only included cases with mWRS greater than zero, i.e., Menière’s disease was still active. This particular cohort of patients presents a challenge in the context of postoperative rehabilitation and programming because of persistent distortions in auditory perception [21]. Fluctuations in speech understanding with CI are to be expected in patients with persistent auditory fluctuations because of active disease. Although long-term care outcomes for inactive Menière’s disease have been described as good [21], active Menière’s still requires considerable clinical or individual resources [22,23]. The impact on WRS_65_(CI) and subjective hearing-related impairment might be substantial [21,22,24]. Previous studies have demonstrated that patients with active Menière’s and fluctuating hearing have increased impedances and require continuous adjustments to the CI sound processor [25]. Kanona et al. [21], suggest that patients with Menière’s disease are likely to require a longer rehabilitation period after cochlear implantation.

### 4.1. Etiology and Modeling

Compared with the differences in WRS_65_(CI) between the individual etiologies and the total population, the model errors for the results of the WRS_65_(CI) for the various etiologies show significantly lower variability (see Table 3). This lower variability suggests that much of the variability, as described by Blamey et al. [15] for the different etiologies, are explained by the preoperatively collected data. In addition, we assume that, especially for the negative-impact etiologies, our patient population represents a positive selection. For example, the included cases of meningitis represent rather mild courses because, as per inclusion criteria, a preoperative mWRS greater than zero was still measurable, and thus no ossification of the cochlea or no or only limited degeneration of the spiral ganglion cells was present. As a rule, CI patients who become deaf following meningitis have worse long-term hearing and speech results [15,26].

We observed the largest negative deviations between measured and predicted WRS_65_(CI), i.e., the largest negative model errors for the etiologies of Menière’s disease, perinatal asphyxia, and trauma, which were accompanied by comparatively higher interquartile ranges of this error. The highest rate of cases missing the prognosis by more than 20 pp was detected in patients with perinatal asphyxia (33%). The lowest deviations in this respect are to be expected for cases with genetic hearing loss, hearing loss, and otosclerosis.

The few patients with a comparatively good 4PTA (<70 dB HL, *n* = 43) and conspicuously low speech understanding represent a constellation that is currently still insufficiently explained. The same applies to cases with very high mWRS, which cannot be approximately achieved with HA at 65 dB SPL. Although we and other clinics [13,14,17,19] can report successful cochlear implantation in this small group of patients, the reasons for this discrepancy between the preoperative unaided pure-tone average hearing threshold and speech understanding remain largely unknown and need to be clarified. There are indications that these cases are to be expected more frequently with increasing age [27]. The objective clarification of these cases appears difficult, as findings that can be clearly interpreted, e.g., via electrocochleography, seem to show a lower incidence with increasing age [28]. Deprivational processes within the auditory periphery offer a possible explanation [29]. Reduced top-down functions, such as impaired linguistic and neurocognitive abilities, should also be considered as a possible cause [30]. To the best of our knowledge, however, no established or scalable methods exist for assessing these functions in routine clinical practice. In summary, despite the currently limited understanding of the pathogenesis and differential diagnosis and the lack of alternative forms of therapy, cochlear implantation is, in the majority of cases, a successful therapy for improving the limited speech understanding obtained with HA preoperatively.

### 4.2. Limits of This Study

In this retrospective study, we were unable to assess the individual WRS_65_(CI) at the six-month time point in all cases suggested by Hoppe et al. [17]. In addition, the COVID pandemic made the scheduled collection of postoperative speech understanding difficult [31]. A meta-analysis of the development of speech understanding showed rapid and significant improvement within the first three months after the first fitting, with no further statistically significant improvement after three months for the average patient [32]. Firszt et al. [16] have also stated that 90% of the final score can be expected after six to seven months. Thus, compared with Hoppe et al. [17], the various measurement times of our work do not bring into question the validity of the mWRS as a minimum predictor, the applicability of the prediction according to Equation (1), or the influence of etiology.

We were also unable to examine the influence of the rehabilitation process on postoperative speech understanding at our clinic, including the sound processor fitting, due to the retrospective study design. It can be assumed that in the case of known comorbidities, there will be greater deviations in the CI rehabilitation and consequently in the WRS_65_(CI). A possible negative influence of comorbidities on postoperative speech understanding could thus be mitigated. A recent study [33] successfully applied the model [17] to systematically relate WRS_65_(CI) deviations from prediction to postoperative audiometry results. By extending the model, the results of Dziemba et al. [33] may offer an explanation for the observed poorer WRS_65_(CI) via significantly poorer audibility in the high-frequency range and possibly insufficient or incorrectly weighted loudness in the different frequency ranges.

Hoppe et al. [19] point out that the prediction via the model or individual deviations from it now influence the processes within postoperative rehabilitation at their clinics. This was not the case in the present retrospective study with cases that were partly treated 18 years ago. In this respect, the number of cases reported here, which miss the prognosis by more than 20 pp, is rather an upper estimate. This means that, fortunately, the prognosis is exceeded by the majority of patients, and only a small proportion of patients do not achieve the prognosis for postoperative speech understanding.

Even though bilateral hearing does play a role in the context of CI provision, this study treated ears separately according to the German CI guidelines and clinical practice [1,5,12,13,14,17,19,34]. To our knowledge, there is no validated model for predicting WRS that can be populated with our baseline audiometric data from the CI ears of both unilateral and bilateral implanted patients. There is a certain but yet unknown variability due to the neglection of contralateral hearing. Within this retrospective study, the corresponding data are not available to a sufficient degree. Further studies are needed to investigate the impact of contralateral hearing loss with respect to outcome prediction.

Further pre- and postoperative studies, including a larger number of patients with rare etiologies and the inclusion of early intervention based on the clear formulation of therapeutic goals for the WRS_65_(CI), therefore seem very reasonable.

## 5. Conclusions

Cochlear implantation of patients with preoperatively measurable speech understanding with optimized HA having sufficient amplification power (HA classified as WHO 4) and a WRS with a HA below 60% represents a promising therapy option. This treatment should even be considered for patients with an average pure tone hearing loss of 60 dB HL (in some individual cases, even below this value) if the fitting of a HA is not successful.

We can confirm the use of preoperative maximum word recognition as a minimum predictor for the postoperative word recognition achievable with CI at 65 dB SPL in our extensive patient population. Moreover, this prediction can be further refined with the model used here. Part of the large interindividual variability in postoperative speech understanding attributable to various etiologies can be explained by the preoperative speech understanding included in the model. For some etiologies, greater variability in outcomes and deviations from prediction have been observed. These should be considered when counseling patients and planning postoperative rehabilitation.

## Figures and Tables

**Table 1:** Demographics of the patient population.

	Male Ears	Female Ears	
Sex	271	330	
Mean age at cochlear implantation [years]	56.2	51.4	
	Mean value	Standard deviation	
Duration of hearing loss [years]	26.3	16.9	
Presumed duration of hearing aid fitting [years]	20.4	14.1	
Implant side	Right	Left	
	290	311	
	Yes	No	Unknown
Tinnitus	363	214	24
Vestibulopathies	98	477	26

**Table 2:** Preoperative four-frequency pure-tone average, 4PTA, and pre- and postoperative word recognition scores.

Preoperative	Minimum	Maximum	Mean	Standard Deviation
4PTA (dB HL)	49.8	120.0	91.0	14.2
mWRS (%)	5.0	100.0	33.2	22.6
WRS_65_(HA) (%)	0.0	60.0	10.4	14.2

**Table 3:** Effect of etiology on word recognition with CI at 65 dB SPL on the model error and its interquartile range.

		Absolute		Relative to Model			
Etiology	Number of Cases	Mean WRS_65_(CI)	Difference to Median WRS_65_(CI)	Median Error [pp]	Adjusted Median Error [pp]	Interquartile Range of Error	Number of Cases Where Prediction Is Missed by More than 20 pp
Genetic hearing loss	14	95	15	15. 5	5.5	17.3	0 (0%)
Sudden hearing loss	66	80	0	11.0	1.0	21.2	3 (5%)
Childhood illness	16	75	−5	10.8	0.9	26.5	3 (20%)
Congenital syndrome	54	75	−5	9.5	−0.5	31.9	10 (19%)
Meningitis	11	80	0	15.0	5.1	14.6	2 (18%)
Ménière’s disease	15	65	−15	−1.8	−11.7	33.7	2 (13%)
Otitis media	23	65	−15	2.2	−7.7	35.7	5 (22%)
Otosclerosis	21	90	10	16.3	6.3	22.7	1 (5%)
Perinatal asphyxia	12	65	−15	−6.8	−16.8	29.7	4 (33%)
Trauma	6	60	−20	−6.2	−16.1	34.0	1 (17%)
Unknown	358	80	0	10.7	0.8	23.1	45 (13%)
Cerebral diseases	5	70	−10	6.2	−3.7	35.8	1 (20%)
Total	601	80	0	9.9	0	17.3	77 (13%)

## Data Availability

Research data are available on request from the last author.

## References

[1] National Institute for Health and Care Excellence (2019). Cochlear Implants for Children and Adults with Severe to Profound Deafness. https://www.nice.org.uk/guidance/ta566.

[2] AWMF (2020). Leitlinien: Cochlea-Implantat Versorgung und Zentral-Auditorische Implantate. https://www.awmf.org/uploads/tx_szleitlinien/017-071l_S2k_Cochlea-Implantat-Versorgung-zentral-auditorische-Implantate_2020-12.pdf.

[3] Van der Straaten T.F.K., Briaire J.J., Vickers D., Boermans P., Frijns J.H.M. (2020). Selection Criteria for Cochlear Implantation in the United Kingdom and Flanders: Toward a Less Restrictive Standard. Ear Hear..

[4] Buchman C.A., Gifford R.H., Haynes D.S., Lenarz T., O’Donoghue G., Adunka O., Biever A., Briggs R.J., Carlson M.L., Dai P. (2020). Unilateral Cochlear Implants for Severe, Profound, or Moderate Sloping to Profound Bilateral Sensorineural Hearing Loss: A Systematic Review and Consensus Statements. JAMA Otolaryngol.-Head Neck Surg..

[5] DGHNO-KHC (2021). Weißbuch Cochlea-Implantat(CI)-Versorgung, 2nd Edition. https://cdn.hno.org/media/2021/ci-weissbuch-20-inkl-anlagen-datenblocke-und-zeitpunkte-datenerhebung-mit-logo-05-05-21.pdf.

[6] Gifford R.H., Dorman M.F., Shallop J.K., Sydlowski S.A. (2010). Evidence for the expansion of adult cochlear implant candidacy. Ear Hear..

[7] Rauch A.K., Metzner T., Aschendorff A., Arndt S., Speck I., Laszig R., Beck R.L. (2019). Speech processor upgrade increases speech comprehension in patients with cochlear implants. HNO.

[8] Wesarg T., Voss B., Hassepass F., Beck R., Aschendorff A., Laszig R., Arndt S. (2018). Speech Perception in Quiet and Noise With an Off the Ear CI Processor Enabling Adaptive Microphone Directionality. Otol. Neurotol..

[9] Aschendorff A., Briggs R., Brademann G., Helbig S., Hornung J., Lenarz T., Marx M., Ramos A., Stöver T., Escudé B. (2017). Clinical investigation of the Nucleus Slim Modiolar Electrode. Audiol. Neurotol..

[10] Aschendorff A., Klenzner T., Arndt S., Beck R., Schild C., Röddiger L., Maier W., Laszig R. (2011). Insertion results for Contour and Contour Advance electrodes: Are there individual learning curves?. HNO.

[11] Hey M., Böhnke B., Mewes A., Munder P., Mauger S.J., Hocke T. (2021). Speech comprehension across multiple CI processor generations: Scene dependent signal processing. Laryngoscope Investig. Otolaryngol..

[12] Hoppe U., Hocke T., Hast AIro H. (2019). Maximum preimplantation monosyllabic score as predictor of cochlear implant outcome. HNO.

[13] Thangavelu K., Nitzge M., Weiß R.M., Mueller-Mazzotta J., Stuck B.A., Reimann K. (2022). Role of cochlear reserve in adults with cochlear implants following post-lingual hearing loss. Eur. Arch. Oto-Rhino-Laryngol..

[14] Rieck J.H., Beyer A., Mewes A., Caliebe A., Hey M. (2023). Extended Preoperative Audiometry for Outcome Prediction and Risk Analysis in Patients Receiving Cochlear Implants. J. Clin. Med..

[15] Blamey P., Artieres F., Başkent D., Bergeron F., Beynon A., Burke E., Dillier N., Dowell R., Fraysse B., Gallégo S. (2013). Factors affecting auditory performance of postlinguistically deaf adults using cochlear implants: An update with 2251 patients. Audiol. Neuro-Otol..

[16] Holden L.K., Finley C.C., Firszt J.B., Holden T.A., Brenner C., Potts L.G., Gotter B.D., Vanderhoof S.S., Mispagel K., Heydebrand G. (2013). Factors affecting open-set word recognition in adults with cochlear implants. Ear Hear..

[17] Hoppe U., Hocke T., Hast A., Iro H. (2021). Cochlear Implantation in Candidates With Moderate-to-Severe Hearing Loss and Poor Speech Perception. Laryngoscope.

[18] Shafieibavani E., Goudey B., Kiral I., Zhong P., Jimeno-Yepes A., Swan A., Gambhir M., Buechner A., Kludt E., Eikelboom R.H. (2021). Predictive models for cochlear implant outcomes: Performance, generalizability, and the impact of cohort size. Trends Hear..

[19] Hoppe U., Hast A., Hocke T. (2023). Validation of a predictive model for speech discrimination after cochlear impIant provision. HNO.

[20] Winkler A., Holube I. (2016). Test-retest reliability of the Freiburg monosyllabic speech test. HNO.

[21] Kanona H., Forde C., Van Rooyen A.M., Keating P., Bradley J., Pendolino A.L., Mehta N., Manjaly J.G., Khalil S., Lavy J. (2022). Cochlear implant outcomes in patients with Meniere’s disease: A large case series. Cochlear Implant. Int..

[22] Hast A., Meßbacher M.E., Liebscher T., Hornung J., Hoppe U. (2021). Fluctuation in electrical hearing in a Morbus Meniere’s patient. Clin. Case Rep..

[23] Pfeiffer C.J., Gehl H.B., Scholtz L.U., Goon P., Sudhoff H., Todt I. (2023). Endolymphatic Hydrops Magnet Resonance Imaging in Ménière’s Disease Patients after Cochlea Implantation. Brain Sci..

[24] Wrobel C., Bevis N.F., Klinge-Strahl A., Strenzke N., Beutner D. (2022). Performance and self-perceived hearing impairment after cochlear implantation in Menière’s disease. Laryngoscope Investig. Otolaryngol..

[25] Samy R.N., Houston L., Scott M., Choo D.I., Meinzen-Derr J. (2016). Cochlear implantation in patients with Meniere’s disease. Cochlear Implant. Int..

[26] Altuntaş O.M., Özkan B., Bajin D., Sennaroğlu G., Sennaroğlu L. (2021). Long-Term Outcome of Cochlear Implantation in Post-meningitic Deafness. J. Int. Adv. Otol..

[27] Hoppe U., Hocke T., Iro H. (2022). Age-Related Decline of Speech Perception. Front. Aging Neurosci..

[28] Riggs W.J., Roche J.P., Giardina C.K., Harris M.S., Bastian Z.J., Fontenot T.E., Buchman C.A., Brown K.D., Adunka O.F., Fitzpatrick D.C. (2017). Intraoperative Electrocochleographic Characteristics of Auditory Neuropathy Spectrum Disorder in Cochlear Implant Subjects. Front. Neurosci..

[29] Walger M., Foerst A., Beutner D., Streicher B., Stürmer K., Lang-Roth R. (2011). Auditory synaptopathy/neuropathy: Clinical findings and diagnosis. HNO.

[30] Moberly A.C., Bates C., Harris M.S., Pisoni D.B. (2016). The Enigma of Poor Performance by Adults With Cochlear Implants. Otol. Neurotol..

[31] Aschendorff A., Arndt S., Kröger S., Wesarg T., Ketterer M.C., Kirchem P., Pixner S., Hassepaß F., Beck R. (2021). Quality of cochlear implant rehabilitation under COVID-19 conditions. HNO.

[32] Ma C., Fried J., Nguyen S.A., Schvartz-Leyzac K.C., Camposeo E.L., Meyer T.A., Dubno J.R., McRackan T.R. (2023). Longitudinal Speech Recognition Changes After Cochlear Implant: Systematic Review and Meta-analysis. Laryngoscope.

[33] Dziemba O.C., Merz S., Hocke T. (2023). Evaluative audiometry after cochlear implant provision. German Version. HNO.

[34] Patro A., Lindquist N.R., Holder J.T., Tawfik K.O., O’Malley M.R., Bennett M.L., Haynes D.S., Gifford R., Perkins E.L. (2022). Further Evidence for Individual Ear Consideration in Cochlear Implant Candidacy Evaluation. Otol. Neurotol..

